# The Role of Nitric Oxide in Mycobacterial Infections

**DOI:** 10.4110/in.2009.9.2.46

**Published:** 2009-04-30

**Authors:** Chul-Su Yang, Jae-Min Yuk, Eun-Kyeong Jo

**Affiliations:** Department of Microbiology and Infection Signaling Network Research Center, College of Medicine, Chungnam National University, Daejeon 301-747, Korea.

**Keywords:** nitric oxide, mycobacteria, macrophages, host defense

## Abstract

Although tuberculosis poses a significant health threat to the global population, it is a challenge to develop new and effective therapeutic strategies. Nitric oxide (NO) and inducible NO synthase (iNOS) are important in innate immune responses to various intracellular bacterial infections, including mycobacterial infections. It is generally recognized that reactive nitrogen intermediates play an effective role in host defense mechanisms against tuberculosis. In a murine model of tuberculosis, NO plays a crucial role in antimycobacterial activity; however, it is controversial whether NO is critically involved in host defense against *Mycobacterium tuberculosis* in humans. Here, we review the roles of NO in host defense against murine and human tuberculosis. We also discuss the specific roles of NO in the central nervous system and lung epithelial cells during mycobacterial infection. A greater understanding of these defense mechanisms in human tuberculosis will aid in the development of new strategies for the treatment of disease.

## INTRODUCTION

Tuberculosis (TB) is a bacterial infectious disease caused by the obligate human pathogen *Mycobacterium tuberculosis* (MTB). TB remains an urgent global health problem, with a third of the global population latently infected and eight million new cases each year. Although only 5~10% of infected individuals develop active TB, the fatality rate is nearly two million people annually (1-3). Following exposure to MTB, a series of immune responses are triggered that ultimately define the course of the infection (4,5). The pathogenesis of infection is complicated; however, recent discoveries have attracted great attention due to their association with host-derived and microbial factors. Advances in free radical research have revealed that the production of reactive oxygen and nitrogen oxide species such as superoxide (O_2_^-^) and nitric oxide (NO) by innate immune cells is a relatively effective host defense mechanism against bacterial, viral, parasitic, and fungal infections (5,6).

The host cells that are protective against TB include macrophages, dendritic cells, T lymphocytes, and alveolar epithelial cells (2,3,7). Macrophages are believed to play a pivotal role in the immune response against mycobacteria through the production of cytokines such as tumor necrosis factor (TNF)-α and interleukin (IL)-1β. TNF-α and IL-1β, along with interferon (IFN)-γ, which is produced by T lymphocytes, can induce NO production in macrophages via the action of inducible forms of the enzyme NO synthase (iNOS) (8-10). NO and related reactive nitrogen intermediates (RNI)s can kill and/or inhibit intracellular pathogens such as mycobacteria (11-14). The actions of iNOS and the production of NO correlate well with antimycobacterial defense in murine models of TB infection (10,12,15). Although it has been demonstrated that iNOS expression is up-regulated in macrophages from human TB lesions (16), few reports have examined the antimycobacterial effects of cytokines and NO released by human macrophages (17,18). These data suggest that human macrophages possess a NO-independent antimicrobial mechanism, although a role for NO in human host defense cannot be excluded.

MTB infects the airways and stimulates alveolar macrophages, epithelial cells, and macrophages. As a result, NO is produced in response to the stimulation of cytokines and chemokines (19). By producing NO, alveolar epithelial cells can actively participate in alveolar inflammatory processes and defense mechanisms against MTB. In this review, we discuss the role of NO in defense mechanisms against MTB and the mechanisms regulating the production of NO in macrophages, including microglia and alveolar epithelial cells.

## OVERVIEW OF NO PRODUCTION AND FUNCTION

NO is a gaseous free radical molecule with pleiotropic functions in pathophysiology that is synthesized by a two-step enzymatic reaction involving a monooxygenase (12,13). One molecule of L-arginine is oxidized at the terminal nitrogen in guanidine to produce *N*^ω^-OH-L-arginine as an intermediate. This intermediate is then further oxidized to form one molecule each of NO and L-citrulline (13,14). L-arginine (a conditionally essential amino acid) is obtained from exogenous (food) and endogenous sources, including whole-body protein degradation and, to a lesser extent, *de novo* synthesis from citrulline by renal arginosuccinate synthase (20,21). Two sequential reactions are catalyzed by NOSs, resulting in the constitutive expression of enzymes primarily in endothelial cells (eNOS) and neuronal cells (nNOS), and as an inducible isoform (iNOS). Constitutively produced NOSs contribute to several physiological processes including vasorelaxation and neurotransmission. In contrast, iNOS is expressed in various cells including macrophages, neutrophils, epithelial cells, and hepatocytes, and it produces excessive NO during infection, inflammation, and states of physiological stimulation (22-24).

**Figure 1:**
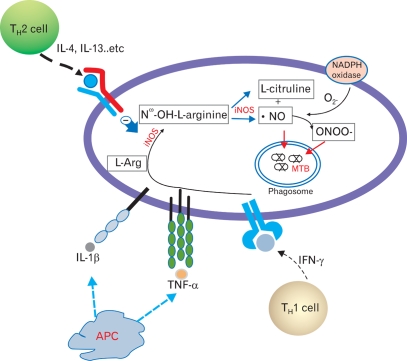
Yang et al. Synthesis, regulation, and antimycobacterial function of NO in mycobacterial infection. Activated inducible nitric oxide synthase (iNOS) produces Nω-OH-L-arginine from L-arginine, and then Nω-OH-L-arginine is transduced to form NO and L-citruline. Synthesis of NO and reactive nitrogen oxides (RNI) are positively regulated by Th1 cytokines, whereas they are negatively regulated by Th2 cytokines. Produced NO and RNIs, which combined with NO and O_2_^-^, can directly kill intracellular MTB in the infected cells (including macrophages, epithelial and glial cells), although the action of NO is dependent on the species and specific cell types.

Th1 cytokines such as IFN-γ, IL-1β, and TNF-α stimulate the expression of macrophage iNOS, leading to NO production. In contrast, under the influence of Th2 cytokines such as IL-4, IL-10, and IL-13, arginine is depleted by arginases (8-10). NO is one of several RNIs with antimicrobial activity (18,25). The increase in RNIs is mediated through reactive nitrogen oxides (e.g., peroxynitrite (ONOO^-^)) generated by the reaction of NO with O_2_^-^ (13,24) (Fig. 1). NO and RNIs can modify bacterial DNA, proteins, and lipids in both the microbe and host. NO can also deaminate and directly damage bacterial DNA by generating abasic sites and strand breaks (7). Other potential killing mechanisms by NO include interactions with accessory protein targets such as iron-sulfur groups, heme groups, thiols, aromatic or phenolic residues, tyrosyl radicals, and amines. These reactions result in enzymatic inactivation and/or other protein malfunctions (26).

## THE ROLE OF NO IN HOST DEFENSE AGAINST MICROBIAL INFECTIONS

During infection with *Mycobacterium*, *Salmonella*, *Streptococcus*, *Leishmania*, or *Bordetella*, excessive NO is produced after the induction of iNOS. In many cases, excessive NO production results in innate resistance to bacterial infection. In a study of *Bordetella pertussis* infection in wild-type (WT) and iNOS-knockout (iNOS KO) mice, the iNOS KO mice displayed increased bacterial growth and susceptibility to infection as compared with the WT mice (27). In a study of murine salmonellosis (*Salmonella typhimurium*), the use of a NO inhibitor, *N*^ω^-monomethyl-L-arginine (L-NMMA), or iNOS KO mice led to similar antimicrobial effects (12,28). In these studies, the lack of NO production was associated with extensive damage, including increased bacterial growth, increased apoptosis, and the exacerbation of histopathological characteristics in mouse livers infected with *Salmonella enterica* serovar Typhimurium (12). Although NO shows antimicrobial activity against bacteria, fungi, and parasites, some studies suggest dual functions during viral infection. NO produced by macrophages and phagocytic cells can act as an effector molecule during innate host defense mechanisms. For example, NO shows antiviral activity in response to certain viruses such as coxsackievirus (29-31), Epstein-Barr virus (32), and herpes simplex virus (HSV)-1 (33-35). In contrast to the antibacterial activity observed with NO, this antiviral activity is associated with nonspecific damage to host cells and tissues, leading to an exacerbation of viral pathogenesis in many infections such as influenza (36), tick-born virus (37), sendai virus (38), HSV-1 (39,40), and cytomegalovirus (41,42). Therefore, despite the antiviral activity of NO, excessive NO production may facilitate viral pathogenesis. These dual functions of NO may lead to differential outcomes during viral infection.

## THE ROLE OF NO IN MYCOBACTERIAL INFECTION: MURINE STUDIES

NO plays a key role in innate immunity and host defense against mycobacteria (1,2,7,43). For example, iNOS KO and immunodeficient mice infected with MTB are at a significantly higher risk of dissemination and mortality as compared with control mice (1,43). In addition, macrophages from mice with the *Bcg/natural resistance associated macrophage protein*-1 resistance phenotype show inhibition of MTB survival through NO production (3,44). Mycobacterial species exhibit variations in susceptibility to NO and its RNIs. For example, murine macrophages have been shown to inhibit the intracellular growth of *M. leprae*, *M. bovis*, and MTB H37Rv (7,11,17,45). When IFN-γ-treated rat alveolar macrophages were infected with *M. avium*, the growth of the bacterium was significantly inhibited by NO synthesized from L-arginine (46).

Contrasting data have been reported in murine and human macrophages infected with *M. avium* (6); neither competitive inhibition by L-NMMA nor depletion of L-arginine by arginase had any effect on *M. avium* growth in murine peritoneal macrophages or human monocyte-derived macrophages (6). In addition, no significant inhibitory effects of NO produced by rat macrophages were observed on the growth of *M. intracellulare* (45). In murine models of latent infection, both NO-dependent (iNOS- and IFN-γ-dependent antimycobacterial mechanisms) and -independent (CD4^+^ T cells required for preventing reactivation of the disease) mechanisms maintain latent TB (4,47); however, the applicability of these reports to humans is uncertain.

## THE ROLE OF NO IN MYCOBACTERIAL INFECTION: HUMAN STUDIES

In contrast to the murine model of TB, there is controversy surrounding the role of NO in the killing and inhibition of MTB in humans (5). The early inhibition of mycobacterial growth by human alveolar macrophages has been shown to be NO-independent (48). Specifically, exogenous IFN-γ failed to produce mycobactericidal effects in human alveolar macrophages (48). Nevertheless, a growing body of evidence suggests that NO production by MTB-infected human monocytes/macrophages, macrophage-like cell lines, and epithelial cells induces mycobacteriostatic activity against MTB (16, 49-52). For example, alveolar macrophages from healthy control subjects infected with MTB produce NO, and this production is correlated with the intracellular inhibition of MTB growth (51). One study demonstrated increased NO production in TB patients as compared with healthy controls following the infection of peripheral blood mononuclear cells (PBMC)s with MTB (50). Other studies have also demonstrated that alveolar macrophages are able to kill mycobacteria and that these antimycobacterial activities are dependent on iNOS expression (49,53). These results suggest a significant role for NO in host defense against mycobacterial infection. Moreover, increased iNOS expression and pulmonary NO production have been reported in alveolar macrophages and PBMCs from TB patients as compared with healthy controls (8,10,16,54). In those studies, NO played a role in the enhancement of TNF-α and IL-1β secretion, which subsequently affected NO production via feedback loops (8). These data indicate an autoregulatory role for NO. It has also been shown that iNOS and nitrotyrosine (a tissue marker of NO metabolism) are expressed in macrophages within granulomatas and areas of TB pneumonitis (15,55). Human PBMCs and bronchial epithelial cells may produce NO when stimulated with MTB-produced NO (50). In addition, the avirulent strain H37Ra was shown to induce significantly higher levels of NO production as compared with the virulent strain H37Rv (50). Recent studies have shown that L-arginine depletion induces the down-regulation of CD3ζ, thereby impairing T cell signaling, whereas the addition of L-arginine leads to CD3ζ re-expression and the recovery of T cell proliferation (18,56). In addition, T cells from TB patients show reduced CD3ζ expression, which is correlated with arginase-induced L-arginine deficiency. These expression levels were normalized with successful TB treatment (21). Taken together, these data suggest that NO plays a contributory role in human host defense against MTB infection.

## THE ROLE OF NO IN MYCOBACTERIAL INFECTION OF THE CENTRAL NERVOUS SYSTEM (CNS)

The roles of NO and iNOS in host defense against infection of the CNS by intracellular pathogens have been reported in previous studies of several intracellular pathogens (e.g., *Toxoplasma gondii* and Sindbis virus) (57,58). The role of microglial cells in neuropathogenesis following CNS infection has been a topic of growing research interest (59). It was previously reported that in contrast to astrocytes, iNOS was not expressed in human microglia following stimulation by IL-1β or IFN-γ (9,60). These findings suggest that NO and iNOS expression may be dependent on cell type and species. Indeed, previous reports demonstrated NO production in activated murine microglia, but not in human microglia (61). Recently, significant effort has been devoted to developing appropriate models of TB infection in the CNS (CNS-TB) using rabbits or mice. Intracerebral inoculation with MTB or *M. bovis* BCG resulted in mononuclear cell infiltration, microglial cell activation, and an increase in the number of bacterial cells within the CNS in a mouse model (62,63). In addition, inoculation with MTB or *M. bovis* BCG led to the up-regulation of IL-1β, TNF-α, IL-6, and IFN-γ within the CNS (63). Recently, Michael et al. (64) reported that iNOS KO mice infected intracerebrally with MTB developed clinical manifestations of CNS-TB, including high mortality rates and histopathological abnormalities resembling human tuberculous meningitis throughout the meninges. The above clinical manifestations were absent in WT mice. These studies underscore the importance of NO in defense against CNS-TB.

## THE ROLE OF NO IN THE MYCOBACTERIAL INFECTION OF EPITHELIAL CELLS

Alveolar epithelial cells are able to actively participate in the pathogenesis of pulmonary inflammatory diseases by producing several cytokines and chemokines (65-67). Alveolar epithelial cells produce NO and various innate immune effectors including chemokines (IL-8), which regulate immune activation. In addition, normal T cells express and secrete RANTES in response to MTB infection (19,67). Strong NO production via iNOS also occurs in human lung epithelial cells (19,66); however, the amount of NO released in response to MTB is not mycobactericidal (65,66). Various cytokines (IFN-γ, TNF-α, and IL-1β; alone or in combination) and mycobacterial components stimulate MTB-infected epithelial cells, inducing NO production and mycobactericidal effects (65,66). These factors may contribute to innate immune control in epithelial cells against intracellular pathogens such as MTB.

## CONCLUDING REMARKS

NO is a nonspecific, chemically reactive molecule that is important in host defense against a wide variety of microbial pathogens. However, it is becoming increasingly clear that specific killing mechanisms and cell types are not sufficient to kill mycobacteria *in vivo*. Although NO is not required for mycobactericidal activity in mouse models, the lack of a role for NO or its products (e.g., ONOO^-^) has not been definitively proven in humans. Nevertheless, a substantial body of evidence indicates a role for NO in human host defenses against MTB. Additional studies are necessary to define the role of NO in relevant human cells including alveolar macrophages, microglia, and epithelial cells. Additionally, it would be useful to generate conditions that mimic *in vivo* environments, such as the co-culture of relevant cells. Such studies, which will refine our understanding of the importance and specific role of NO in TB defense, may lead to innovative strategies for TB treatment.
